# Microbial Quality of Donkey Milk during Lactation Stages

**DOI:** 10.3390/foods12234272

**Published:** 2023-11-26

**Authors:** Miaomiao Zhou, Fei Huang, Xinyi Du, Changfa Wang, Guiqin Liu

**Affiliations:** School of Agricultural Science and Engineering, Liaocheng Research Institute of Donkey High-Efficiency Breeding and Ecological Feeding, Liaocheng University, Liaocheng 252000, Chinawangchangfa@lcu.edu.cn (C.W.); guiqinliu@lcu.edu.cn (G.L.)

**Keywords:** microbiota composition, donkey milk, 16S rRNA, lactation stages

## Abstract

The microbial community in donkey milk and its impact on the nutritional value of donkey milk are still unclear. We evaluated the effects of different lactation stages on the composition and function of donkey milk microbiota. The milk samples were collected at 1, 30, 60, 90, 120, 150, and 180 days post-delivery. The result showed that the microbial composition and functions in donkey milk were significantly affected by different lactation stages. The dominant bacterial phyla in donkey milk are *Proteobacteria* (60%) and *Firmicutes* (22%). *Ralstonia* (39%), *Pseudomonas* (4%), and *Acinetobacter* (2%) were the predominant bacterial genera detected in all milk samples. In the mature milk, the abundance of lactic acid bacteria *Streptococcus* (7%) was higher. *Chloroplast* (5%) and *Rothia* (3%) were more plentiful in milk samples from middle and later lactation stages (90–180 d). Furthermore, the pathogens *Escherichia-Shigella* and *Staphylococcus* and thermoduric bacteria *Corynebacterium*, *Arthrobacter*, and *Microbacterium* were also detected. Donkey milk is rich in beneficial bacteria and also poses a potential health risk. The above findings have improved our understanding of the composition and function changes of donkey milk microbiota, which is beneficial for the rational utilization of donkey milk.

## 1. Introduction

Donkey milk (DM) is rich in lactose and low in fat, which is closer to human milk than dairy milk [1]. Some research has indicated that DM is an ideal substitute for infants with milk protein allergy and is also ideal nutrition for growing children, convalescent patients, and the elderly [2,3,4]. In recent years, DM has gradually been considered and recognized by consumers. In 2019, the output of DM in China was about 270,000 tons, with an output value of nearly CNY 1 billion [5].

DM has lots of important bioactivities, such as immune stimulation, anti-inflammatory activity, antioxidant activity, and antibacterial and anti-virus effects. Some research showed that DM has anti-inflammatory and immune-stimulating effects and is effective in the treatment of diseases such as diabetes, tuberculosis, and cancer [6,7,8]. Proteomic analysis of DM showed that lysozyme, lactoferrin, immunoglobulin, and other whey proteins had antibacterial activity [3]. Due to the high concentration of antibacterial factors such as lysozyme and lactoferrin, the microbial population in DM is relatively low [4].

There are many studies on the nutritional characteristics of DM, but there is limited research on the microbial community in DM. In a study by Colavita et al. (2016), they found both the microbial contamination and somatic cell count in DM were reasonably low. However, there are also pathogens in DM, such as *Escherichia coli* O157 and *Salmonella* spp. [9]. In a study by Soto Del Rio et al. (2016), microorganisms that may have important probiotic activity were also found in DM [10]. Derdak et al. (2021) isolated and identified beneficial bacteria in DM and evaluated their antibacterial activity against pathogenic bacteria such as *Staphylococcus aureus* [11]. Furthermore, the microbial composition in DM is complex, and its distribution between and within farms is variable [10,12,13].

Polymerase chain reaction (PCR) technology is widely used for food bacteria detection. Niamah (2012) detected Listeria monocytogenes in milk with the PCR method [14]. Another study used multiple polymerase chain reaction (mPCR) to simultaneously detect several pathogens in milk [15]. With the development of DNA sequencing technology, high-throughput sequerncing (HTS) technology has been developed and widely used for bacterial detection in milk [12,13].

There are few studies on the changes in microbiota in DM over time. The lactation stage may alter the composition of the microbiota in DM, thereby affecting its functional properties. Thus, in order to evaluate the microbial quality of DM and explore and utilize beneficial bacteria in DM, the DM microbial diversity and community throughout lactation to 180 days were investigated with the HTS method in this study.

## 2. Materials and Methods

### 2.1. Collection and Preparation of DM

Milk samples were collected from 35 healthy Dezhou donkeys, with 5 per lactation stage: 1 d (group A), 30 d (group B), 60 d (group C), 90 d (group D), 120 d (group E), 150 d (group F), and 180 d (group G) after foaling. The donkeys were housed at a farm in Liaocheng City, Shandong Province, China. The donkeys were raised in a semi-closed house. All donkeys were offered water and the same diet of grass hay as desired supplemented with 2 kg concentrate/head/day. The concentrate consisted of corn, soybean meal, expanded soybeans, bran, mineral additives, vitamin additives, and amino acid additives. The milk samples were collected during morning mechanical milking, frozen quickly with liquid nitrogen and stored at −80 °C. The animal experiment of this study was approved by the Animal Care and Use Committee of Liaocheng University (Shandong, China) (2023022706).

### 2.2. Sequencing

#### 2.2.1. DNA Extraction

CTAB method was used in total genome DNA extraction. Briefly, we added 200 μL of milk sample to 1000 μL of CTAB lysate containing lysozyme, performed a 65 °C water bath, and then centrifuged the mixture at 12,000 rpm for 10 min. Further, we added an equal volume of phenol, chloroform, and isoamyl alcohol (25:24:1) to the supernatant and centrifuged it at 12,000 rpm for 10 min. After that, we added an equal volume of chloroform and isoamyl alcohol (24:1) to the supernatant and centrifuged it at 12,000 rpm for 10 min. Then, we added 3/4 volume of isopropanol to the supernatant to precipitate DNA, centrifuged it at 12,000 rpm for 10 min, and washed the DNA twice with 75% ethanol. Finally, we added sterile water to dissolve the DNA sample. To detect DNA purity, 1% agarose gel electrophoresis was used (100 volts, 40 min). The DNA contents were measured by ultraviolet spectrophotometry. Then, we diluted the DNA with sterile water to 1 ng/µL.

#### 2.2.2. Amplification of 16S rRNA and Detection of PCR Products [16]

Specific primers (515F: 5′-CCTAYGGGRBGCASCAG-3′; 806R: 5′-GGACTACNNGGGTATCTAAT-3′) (Sangon Biotech, Shanghai, China) and barcodes were used to amplify the 16S rRNA gene in V3–V4 regions. We performed the PCR reaction on a Bio-rad T100 gradient PCR instrument (Bio-rad, Hercules, CA, USA). The PCR reaction mixture included Phusion^®^ High-Fidelity PCR Master Mix (New England Biolabs, Ipswich, MA, USA) (15 µL), primer (0.2 µM), and DNA (10 ng). The cycling conditions were as follows: 98 °C for 1 min, then cycling 30 times at 98 °C (10 s), 50 °C (30 s), 72 °C (30 s), and, finally, 72 °C for 5 min. We mixed an equal volume of 1X loading buffer (containing SYB green) to the PCR products and used 2% agarose gel electrophoresis to detect DNA (80 volts, 40 min). We mixed the PCR products in equal proportion and then used the Qiagen Gel Extraction Kit (Qiagen, Hilden, Germany) to purify the mixed PCR products.

#### 2.2.3. Library Preparation and Sequencing

Sequencing libraries were generated with the NEBNext^®^ Ultra™ IIDNA Library Prep Kit (cat. no. E7645) according to the manufacturer’s recommendations. The Qubit^@^ 2.0 Fluorometer (Thermo Scientific, Waltham, MA, USA) and Agilent Bioanalyzer 2100 system were used to evaluate the library quality. Finally, the library was sequenced on the Illumina NovaSeq platform, and a paired-end reading of 250 bp was generated.

### 2.3. Data Analysis

#### 2.3.1. Paired-End Reads Merged and Quality Control

Paired-end readings were assigned to samples based on their unique barcodes and truncated by cutting off the barcodes and primer sequences. Paired-end reads were merged using FLASH (version 1.2.11, http://ccb.jhu.edu/software/FLASH/ (accessed on 7 December 2022)) [17], and the splicing sequences were called Raw Tags. Then, we used Fastp (version 0.20.0) software to filter the quality of the Raw Tags to obtain high-quality Clean Tags. We integrated Clean Tags into the reference database (Silva database https://www.arb-silva.de/ (accessed on 7 December 2022)) to detect chimeric sequences using Vsearch (version 2.15.0) and removed them to obtain the Effective Tags [18].

#### 2.3.2. ASVs Denoise and Species Annotation

For the previously obtained Effective Tags, we used the DADA2 or deblur module of QIIME2 software (version QIIME2-202006) for denoising to obtain the initial ASVs (Amplicon Sequence Variants) (default: DADA2) and then filter out ASVs with an abundance of less than 5 [19]. Species annotation was conducted using QIIME2 software with the Silva database as the annotation database. In order to study the phylogenetic relationships of each ASV and the differences in dominant species among different groups, multiple sequence alignments were conducted using QIIME2 software. We normalized the absolute abundance of ASVs using the sequence number standard corresponding to the sample with the lowest sequence. Subsequent analyses of alpha diversity and beta diversity were all performed based on output normalization data.

#### 2.3.3. Alpha Diversity

In order to analyze the diversity, richness, and uniformity of the communities in the sample, alpha diversity was calculated from 7 indices (Observed_otus, Chao1, Shannon, Simpson, Dominance, Good’s coverage, and Pielou_e) in QIIME2 [20].

#### 2.3.4. Beta Diversity

To evaluate the complexity of community composition and compare the differences between groups, the weighted and unweighted UniFrac distances in QIIME2 were used to calculate beta diversity [21,22,23].

Cluster analysis was conducted using principal component analysis (PCA), which was used to reduce the dimensionality of the original variables using the ade4 package and ggplot2 package in R software (version 3.5.3).

Principal coordinate analysis (PCoA) was used to obtain the principal coordinates and visual differences of samples in complex multi-dimensional data. We converted the matrix of weighted or unweighted uniform distances among previously obtained samples into a new set of orthogonal axes where the maximum variation factor was represented by the first principal coordinate, the second maximum variation factor was represented by the second principal coordinate, and so on. We used the QIIME2 package to display three-dimensional PCoA results, while we used the ade4 package and ggplot2 package in R software (version 2.15.3) to display two-dimensional PCoA results.

In order to study the significance of community structure differences between groups, the adonis and anosim functions in QIIME2 software were used for analysis. To identify significantly different species at each taxonomic level (phylum, class, order, family, genus, species), MetaStat and *t*-test analyses were conducted using R software (version 3.5.3). LEfSe software (version 1.0) was used to perform LEfSe analysis (LDA score threshold: 4) to identify biomarkers.

In addition, in order to study the functions of communities in the sample and identify the different functions of communities in different groups, PICRUSt2 software (version 2.1.2-b) was used for functional annotation analysis.

#### 2.3.5. Statistical Analysis

The *t*-test was used for community structure and functions of communities analysis. *p* < 0.05 was defined as a significant difference.

## 3. Results and Discussion

### 3.1. Diversity of Microbiota in DM from Different Lactation Stages

**Figure 1:**
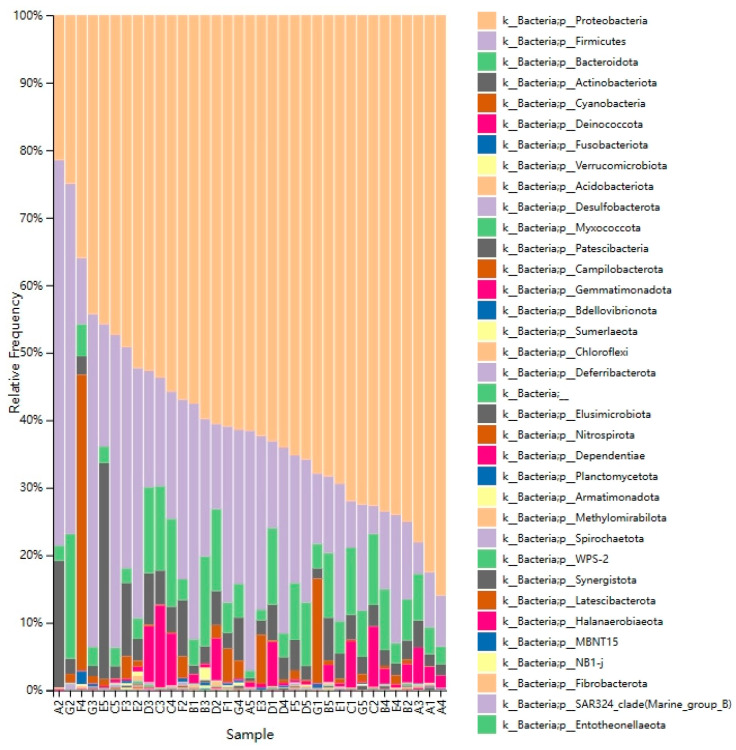
Relative frequency of bacterial phyla in donkey milk. Milk samples were collected from Dezhou donkeys at 7 different lactation stages: 1 d (A), 30 d (B), 60 d (C), 90 d (D), 120 d (E), 150 d (F), and 180 d (G) after foaling.

**Figure 2:**
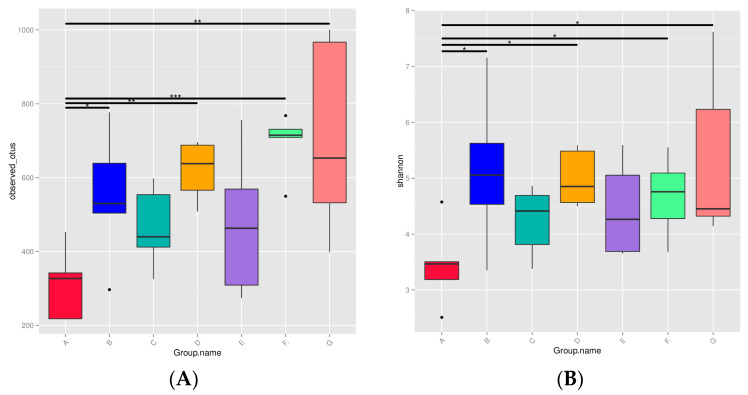
The bacterial alpha diversity ((**A**): observed OTUs; (**B**): Shannon index) in donkey milk. Milk samples were collected from Dezhou donkeys at 7 different lactation stages: 1 d (A), 30 d (B), 60 d (C), 90 d (D), 120 d (E), 150 d (F), and 180 d (G) after foaling. The microbiota richness and diversity were significantly higher in milk samples of groups B, D, F, and G (mature milk) than in the colostrum (group A) (Figure 2). Wilcoxon test was used for data statistical analysis. The median values are represented by the horizontal bars within the boxes. The “*” indicated that 0.01 < *p* < 0.05; The “**” indicated that 0.001 < *p* < 0.01. The “***” indicated that *p* < 0.001. The black dots in the figure indicated outliers.

The relative abundance of bacterial phyla in 35 different DM samples is shown in Figure 1. The relatively abundant bacteria in DM are mainly *Proteobacteria* (60%), *Firmicutes* (22%), *Bacteroidota* (7%), *Actinobacteriota* (5%), *Cyanobacteria* (3%), and *Deinococcota* (2%). The bacterial alpha diversity in DM is showed in Figure 2. The observed OTUs and Shannon index were significantly different between the milk from different lactation stages (Figure 1). Both observed OTUs (Wilcoxon; *p*: B-A = 0.0297, C-A = 0.1646, D-A = 0.0051, E-A = 0.1175, F-A = 4 × 10^−4^, G-A = 0.0022) and Shannon index (Wilcoxon; *p*: B-A = 0.014, C-A = 0.1953, D-A = 0.0102, E-A = 0.1083, F-A = 0.0398, G-A = 0.0191) indicated that the microbiota richness and diversity were greater in milk samples of groups B, D, F, and G than in the colostrum (group A) (Figure 2).

**Figure 3:**
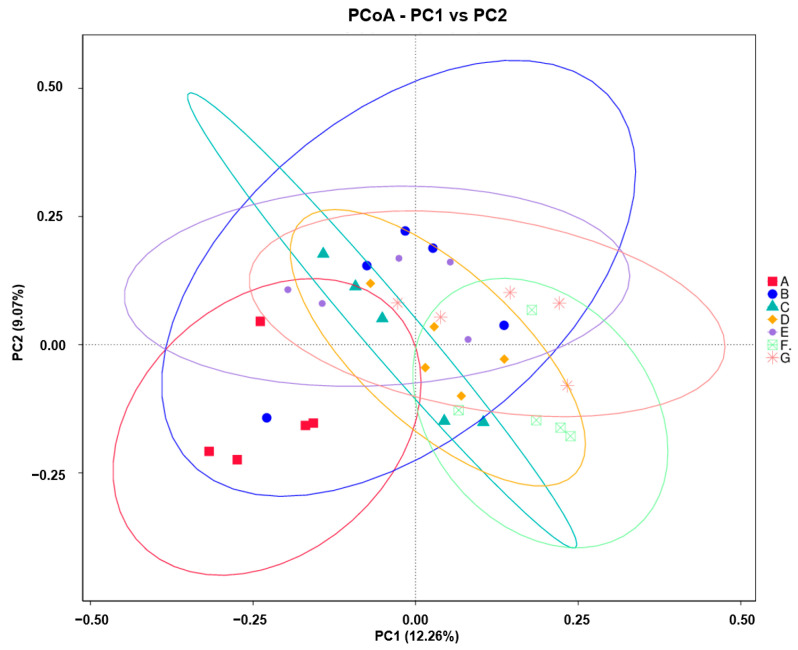
The microbial beta diversity of donkey milk of different lactation stages. The beta diversity was represented using the principal coordinate analysis (PCoA). Milk samples were collected from Dezhou donkeys at 7 different lactation stages: 1 d (A), 30 d (B), 60 d (C), 90 d (D), 120 d (E), 150 d (F), and 180 d (G) after foaling. The microbiota of each donkey milk sample is represented by a point on the PCoA diagram. The samples clustered together have the most similar microbiota. The first coordinate explains 12.26%, and the second coordinate explains 9.07% of variation between mature milk (groups B, C, D, E, F, G) and colostrum (group A) samples.

The principal coordinate analysis (PCoA) representation of the beta diversity analysis was used to compare bacterial communities in milk samples of different lactation stages. Moderate clustering of samples based on different lactation stages in the PCoA diagram is shown in Figure 3. Based on the *t*-test, there were significant differences in microbial beta diversity between any two groups (A, B, C, D, E, F, and G) of DM, except for groups C and E, E and G, F and G.

### 3.2. Microbes Numbers in DM of Different Lactation Stages

**Figure 4:**
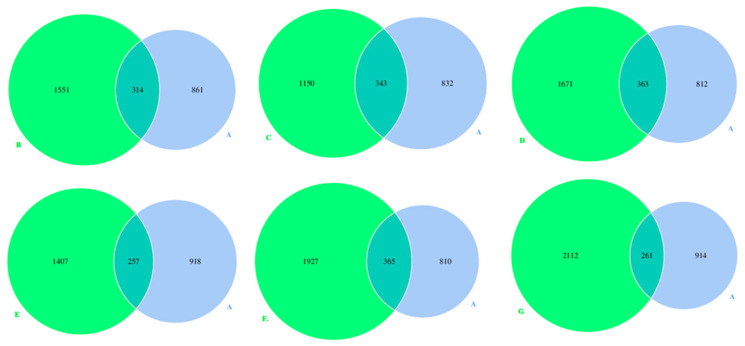
Venn diagrams created using the data of the bacterial OTUs (operational taxonomic units) in donkey milk of different lactation stages. Milk samples were collected from Dezhou donkeys at 7 different lactation stages: 1 d (A), 30 d (B), 60 d (C), 90 d (D), 120 d (E), 150 d (F), and 180 d (G) after foaling.

The Venn diagrams were created using the data of the most prevalent OTUs, which showed the microbe numbers and differences of each milk sample at different lactation stages (Figure 4). The number of OTUs shared by group A and groups B, C, D, E, F, and G was 314, 343, 363, 257, 365, and 261, respectively, representing 11.5%, 14.8%, 12.8%, 10.0%, 11.8%, and 7.9% of all the OTUs.

### 3.3. Microbiota Composition in DM of Different Lactation Stage

**Figure 5:**
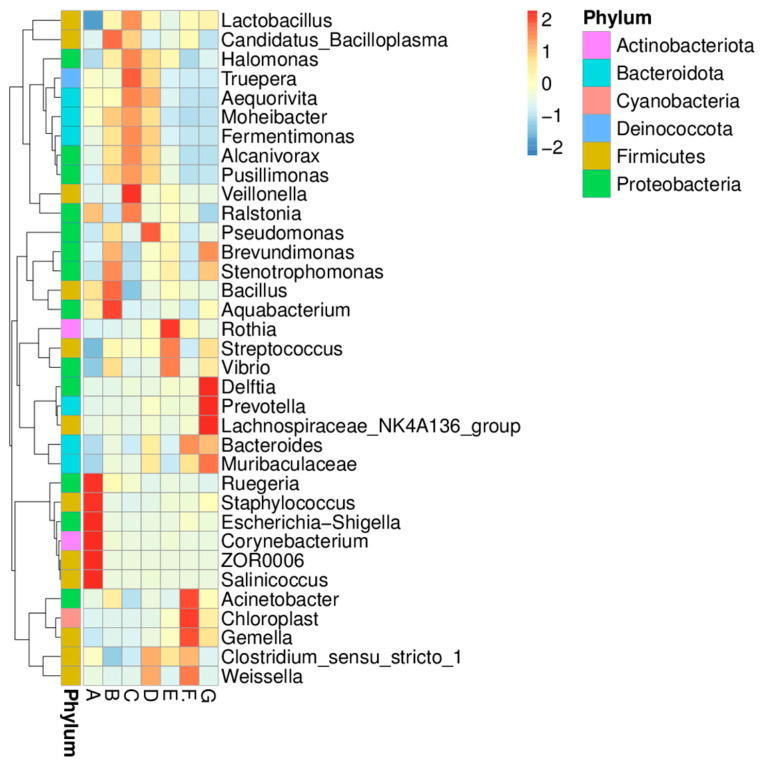
The relative abundances of the bacterial genera (top 35) in donkey milk of seven lactation stages. Milk samples were collected from Dezhou donkeys at 7 different lactation stages: 1 d (A), 30 d (B), 60 d (C), 90 d (D), 120 d (E), 150 d (F), and 180 d (G) after foaling. Red (0–2) indicates genera with relatively high values, while blue (0–(−2)) indicates genera with lower values. Significant differences in the composition of microbiota at the genus level were detected in donkey milk of different lactation stages.

**Figure 6:**
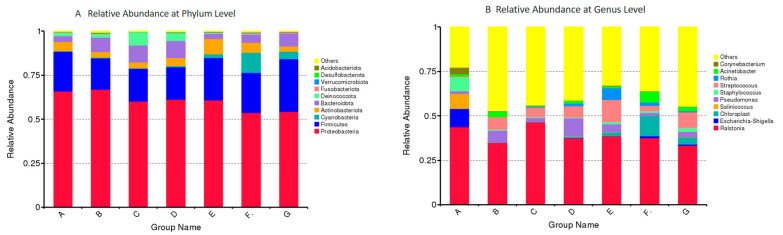
The relative abundances of bacterial phyla (**A**) and genera (**B**) (top 10). Milk samples were collected from Dezhou donkeys at 7 different lactation stages: 1 d (A), 30 d (B), 60 d (C), 90 d (D), 120 d (E), 150 d (F), and 180 d (G) after foaling.

The microbiota compositions and the bacteria relative abundance of DM samples from different lactation stages were analyzed. The relative abundances of the bacterial genera (top 35) in DM of seven lactation stages are shown in Figure 5. The relative abundances of phyla and genera (top 10) are shown in Figure 6. The *Proteobacteria* and *Firmicutes* had high levels in the DM. The *Ralstonia* (39%), *Pseudomonas* (4%), and *Acinetobacter* (2%) were the predominant bacterial genera detected in all milk samples. *Streptococcus* (7%) was more abundant in the mature milk (B, C, D, E, F, G)*. Rothia* (3%) and *Chloroplast* (5%) were more abundant in milk samples from the middle and later lactation stages (D, E, F, G). Furthermore, the other predominant bacterial genera detected in group A were *Escherichia-Shigella* (10%), *Salinicoccus* (9%), *Staphylococcus* (8%), and *Corynebacterium* (4%).

**Figure 7:**
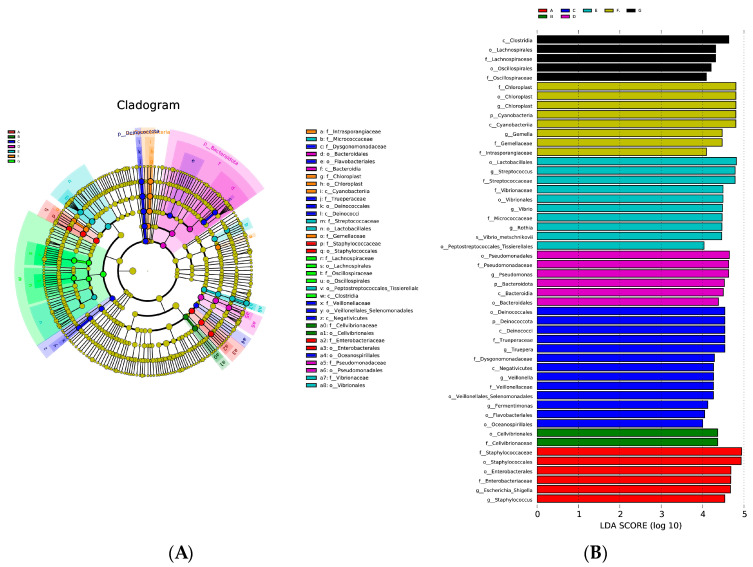
Donkey milk microbiota taxonomic differences at different lactation stages analyzed using LEfSe (linear discriminant analysis effect size). Milk samples were collected from Dezhou donkeys at 7 different lactation stages: 1 d (A), 30 d (B), 60 d (C), 90 d (D), 120 d (E), 150 d (F), and 180 d (G) after foaling. Picture (**A**) is the cladogram of the differential milk microbial taxa. Picture (**B**) is the histograms of linear discriminant analysis scores of 16S gene sequences with a cut off value of LDA (linear discriminant analysis) score > 4 (log10).

The microbial composition and community structure of DM samples of different lactation stages were analyzed for significant differences with LEfSe (linear discriminant analysis effect size). When the log linear discriminant analysis (LDA) score > 4.0 and *p* < 0.05 (Kruskal–Wallis test and Wilcoxon test), the difference was significant. The results showed that there were significant microbiota taxonomic differences among the DM from the seven lactation stages (A, B, C, D, E, F, G) (6, 2, 13, 6, 10, 8, 5 phylotypes) (*p* < 0.05) (Figure 7). In colostrum (group A), *Escherichia-Shigella* and *Staphylococcus* were the dominant bacterial genera, while, in milk group C, *Truepere*, *Fermentimonas*, and *Veillonella* were dominant, in milk group D, *Pseudomonas* was dominant, in milk group E, *Streptococcus*, *Rothia*, and *Vibrio* were dominant, and, in milk group F, *Chloroplast* and *Gemella* were dominant.

### 3.4. Functional Differences of Microbiome in DM of Different Lactation Stages

**Figure 8:**
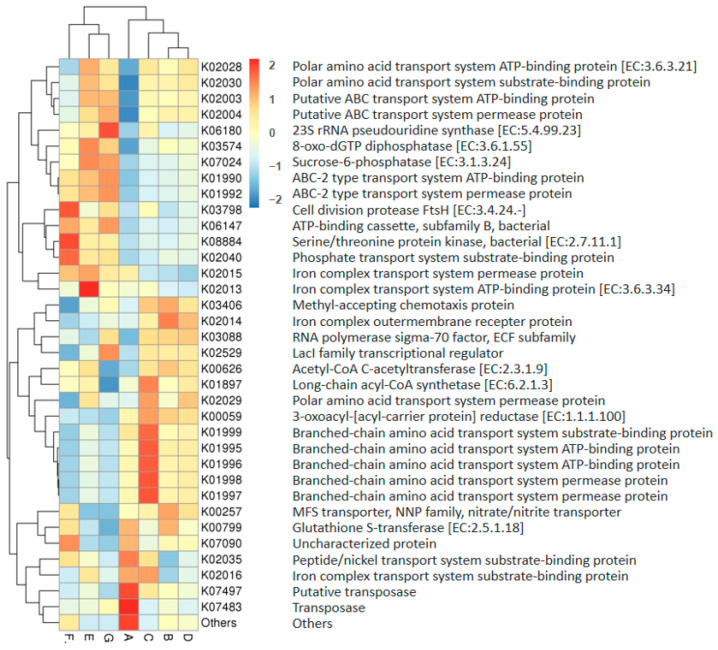
The relative abundance of the top 35 microbiota metabolic functions in donkey milk of different lactation stages. Milk samples were collected from Dezhou donkeys at 7 different lactation stages: 1 d (A), 30 d (B), 60 d (C), 90 d (D), 120 d (E), 150 d (F), and 180 d (G) after foaling. Red indicates functions with relatively high values (0–2), while blue represents a relatively low value (0–(−2)).

The metabolic functions of microbiota in DM were predicted by the PICRUSt2 program (Phylogenetic Investigation of Communities by Reconstruction of Unobserved States) according to the bacterial 16S rRNA sequencing data. The molecular function was characterized by analyzing the KEGG Orthology (KO, https://www.genome.jp/kegg/ko.html (accessed on 7 December 2022)) database. The relative abundance of the top 35 metabolic functions in DM from different lactation stages is shown in Figure 8. DM microbiota functions were significantly different in DM of different lactation stages. The significantly different metabolic functions between mature DM (B, C, D, E, F, G) and colostrum (A) are shown in Table 1.

**Figure 9:**
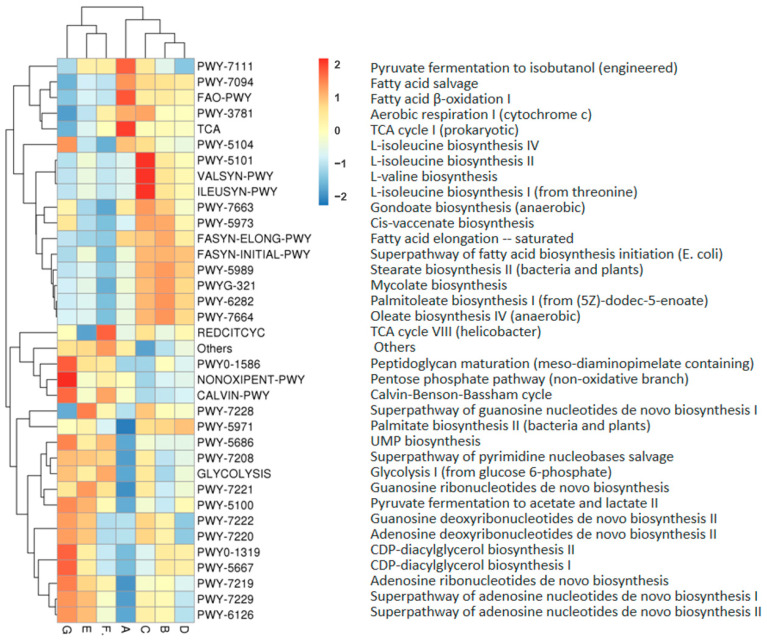
The pathways related to the microbiota differential metabolic functions. Milk samples were collected from Dezhou donkeys at 7 different lactation stages: 1 d (A), 30 d (B), 60 d (C), 90 d (D), 120 d (E), 150 d (F), and 180 d (G) after foaling. Red indicates pathways with relatively high values (0–2), while blue represents a relatively low value (0–(−2)).

The pathways related to the microbiota differential metabolic functions are displayed in Figure 9. TCA cycle I (prokaryotic), fatty acid beta-oxidation I, and pyruvate fermentation to isobutanol (engineered) were the significantly enriched pathways in milk of group A. Oleate biosynthesis IV (anaerobic), mycolate biosynthesis, palmitoleate biosynthesis I (from (5Z)-dodec-5-enoate), and stearate biosynthesis II (bacteria and plants) were significantly higher in milk from group B. In milk from group C, L-valine biosynthesis, L-isoleucine biosynthesis II, and L-isoleucine biosynthesis I (from threonine) were significantly enriched pathways. In milk of group D, the pathway for palmitate biosynthesis II (bacteria and plants), the superpathway of fatty acid biosynthesis initiation (*E. coli*), and stearate biosynthesis II (bacteria and plants) were much more abundant. The relatively abundant of pathways, the superpathway of guanosine nucleotides de novo biosynthesis and guanosine ribonucleotides de novo biosynthesis of milk, from group E were significantly higher than for other groups. The relatively abundant pathways TCA cycle VIII (helicobacter) and glycolysis I (from glucose 6-phosphate) in milk from group F. were significantly higher than for other groups. Significantly enriched pathways of milk from group G were the pentose phosphate pathway (non-oxidative branch), peptidoglycan maturation (meso-diaminopimelate containing), the Calvin–Benson–Bassham cycle, CDP-diacylglycerol biosynthesis II, and CDP-diacylglycerol biosynthesis I.

## 4. Discussion

DM is a characteristic, high-quality variety of milk source, and its nutritional composition is very close to that of human milk. DM has a low fat content and a high proportion of unsaturated fatty acids. Compared with dairy milk, DM reduces calorie intake and the risk of diseases such as hyperlipidemia, which is conducive to cardiovascular health [4,24]. In addition, DM contains a variety of functional nutritional factors such as lysozyme, lactalbumin, lactoferrin, and immunoglobulin [2,25,26]. DM can improve glucose and lipid metabolism and detoxification activities in rats by regulating the gut microbiota (affecting the proportions of bacterial phyla and genera) [27]. Research has found that DM can affect the gut microbiota composition of donkey foals. As foals shift from milk consumption to a forage and grain diet, there are identifiable changes in the fecal microbial composition of their feces [28]. A properly established gastrointestinal microbiota is beneficial for the healthy growth of foals, and abnormalities in the microbiota can affect the health of foals and cause diseases such as diarrhea [29].

The yield of DM and the content of milk protein, lactose, and milk fat vary with different lactation stages [29,30,31]. There is little research on the changes in the microbial composition of DM during lactation. The microbial composition and function in DM of different lactation stages were investigated via 16S rRNA high-throughput sequencing technology in this study. The results indicated that the relatively abundant bacterial phyla in DM are mainly *Proteobacteria* and *Firmicutes*, followed by *Bacteroidota*, *Actinobacteriota*, *Cyanobacteria*, and *Deinococcota*. This result is in-keeping with the conclusion drawn by Luoyizha et al. (2020) for DM [13]. The microbial composition in milk is complex and influenced by many factors. Research has shown that lactation stage is the main factor causing human milk microbiota composition changes [32]. The bacterial composition of goat colostrum has significant differences compared to that of mature milk [16]. Our findings suggest that the lactation stage is an important factor affecting the composition of DM microbiota. There were significant differences in bacterial and functional compositions in different groups of DM. The *Ralstonia*, *Pseudomonas*, and *Acinetobacter* were the predominant bacterial genera detected in all milk samples. *Streptococcus* was more abundant in the mature milk. *Rothia* and *Chloroplast* were more abundant in milk samples from the middle and later lactation stages (90–180 d). Furthermore, the other predominant bacterial genera detected in group A were *Escherichia-Shigella*, *Salinicoccus*, *Staphylococcus*, and *Corynebacterium*. In addition, the breeding environment is another key factor affecting the microbial composition in milk. Luoyizha et al. (2020) found a significant difference in genera richness between DM samples from two different places in China [13]. The distribution of bacteria and biotypes in DM between and within farms is variable [10,12]. A study conducted in Italy showed that the bacteria genera with higher relative abundance in DM were *Pseudomonas* and *Ralstonia*. Another study in Italy found that *Pseudomonas* and *Chryseobacterium* were the main bacterial genera in DM. However, in a study from Cyprus, the *Sphingomonas*, *Mesorhizobium*, and *Pseudomonas* were detected to have relatively high abundance in DM [4]. It can be seen that *Pseudomonas* is the most common bacterial genus in DM.

Antimicrobial factors can promote the intestinal beneficial microbiota growth, thereby eliminating pathogens and preventing infection [33]. Probiotics such as *Lactobacillus* and *Lactococcus* have been used for mastitis treatment and have achieved good therapeutic effects [34]. Microorganisms that may have important probiotic activity have also been found in DM. Derdak et al. (2021) isolated and identified lactic acid bacteria in DM and evaluated their antibacterial activity against pathogenic bacteria such as *Staphylococcus aureus* [11]. It was found that *Carnobacterium*, *Enterococcus*, *Lactobacillus*, *Lactococcus*, *Leuconostoc*, and *Streptococcus* were present in DM [4,10,12]. In this study, all the above lactic acid bacteria were also found in DM. The lactic acid bacteria were mainly composed of *Streptococcus* and *Lactobacillus*, with a relatively low abundance of *Enterococcus*, *Leuconostoc*, *Lactococcus*, and *Carnobacterium*. The *Streptococcus*, *Lactobacillus*, *Enterococcus*, and *Leuconostoc* were more abundant in mature milk, while *Lactococcus* and *Carnobacterium* had relatively high content in colostrum. Therefore, DM is a good source of beneficial bacteria. An appropriate number of beneficial bacteria is beneficial for the immune system development of the intestine and can make sure that infants experience healthy growth [35].

However, there are also pathogens in DM, such as *Escherichia coli O157*, *Salmonella spp*., and *Listeria monocytogenes* [4,9]. Furthermore, some thermoduric bacteria (*Bacillus*, *Arthrobacter*, *Microbacterium*, and *Corynebacterium*) are reported to be present in DM [13]. In this study, both the pathogens *Escherichia-Shigella* and *Staphylococcus* and thermoduric bacteria *Corynebacterium*, *Arthrobacter*, and *Microbacterium* were detected in DM. This could cause sterilization failure during thermal processing or cause diseases. Moreover, the *Escherichia-Shigella*, *Staphylococcus*, and *Corynebacterium* were predominant bacterial genera detected in colostrum at significantly higher levels than in mature milk. It is speculated that this may be related to antibacterial components such as lysozyme and lactoferrin in milk. The lysozyme content of raw DM is high, which is the main factor that exerts antibacterial activity, so it can keep a low level of bacteria in DM and a long natural shelf life [36]. In another study, we found the relative expression level of lysozyme and lactoferrin in mature milk was significantly higher than that in colostrum (unpublished data). This may be the reason for the low abundance of pathogens in mature milk, but further research is still needed.

Analysis of the metabolic functions of microbiota revealed significant differences in the function of microbiota in DM at different lactation stages. The significantly enriched pathways (TCA cycle I, fatty acid beta-oxidation I, and pyruvate fermentation to isobutanol) in colostrum were mainly related to energy generation. In the early lactation stage, the metabolic pathways of unsaturated fatty acid synthesis (30 d, oleate and palmitoleate) and amino acids synthesis (60 d, L-valine and L-isoleucine) were more active. In the middle lactation stage, the metabolic pathways regulating saturated fatty acid synthesis (90 d, palmitate and stearate) and nucleotide synthesis (120 d, guanosine nucleotides) were significantly enriched in DM. In the late lactation stage, the enriched metabolic pathways were related to energy production (150 d, TCA cycle and glycolysis) and the synthesis of functional biomolecules (180 d, pentose phosphate pathway, peptidoglycan maturation, and CDP-diacylglycerol). The above results suggest that the lactation stage may affect the DM function by changing the composition of microorganisms. The mechanism by which microbiota affect the function of DM needs further research.

## 5. Conclusions

In conclusion, the dominant bacterial phyla in DM were *Proteobacteria* and *Firmicutes*; the *Ralstonia*, *Pseudomonas*, and *Acinetobacter* were the predominant bacterial genera detected in DM; there were beneficial bacteria, pathogens, and thermoduric bacteria in DM; and the microbiota composition and function altered with the lactation stage. The above findings have improved our understanding of the microbial quality of DM during lactation stages, which is beneficial for the rational utilization of DM. Further research is needed on the microbial quality evaluation of DM, the utilization of beneficial bacteria in DM, and the effects of antibacterial components on microorganisms in DM, as well as the relationship between microbial function and DM function.

## Figures and Tables

**Table 1:** The significantly different metabolic functions between colostrum (A) and mature donkey milk (B, C, D, E, F, G) (*t*-test, *p* < 0.05).

	KO_Hierarchy	KEGG_Description	*p* Value
A vs. B	K02004	putative ABC transport system permease protein	0.02
	K02529	LacI family transcriptional regulator	0.021
	K02495	oxygen-independent coproporphyrinogen III oxidase (EC:1.3.98.3)	0.038
	K02342	DNA polymerase III subunit epsilon (EC:2.7.7.7)	0.030
A vs. C	K02003	putative ABC transport system ATP-binding protein	0.026
	K02004	putative ABC transport system permease protein	0.014
	K02529	LacI family transcriptional regulator	0.028
	K15634	probable phosphoglycerate mutase (EC:5.4.2.12)	0.012
	K02342	DNA polymerase III subunit epsilon (EC:2.7.7.7)	0.025
A vs. D	K02003	putative ABC transport system ATP-binding protein	0.015
	K02004	putative ABC transport system permease protein	0.006
	K02529	LacI family transcriptional regulator	0.004
	K15634	probable phosphoglycerate mutase (EC:5.4.2.12)	0.011
	K02342	DNA polymerase III subunit epsilon (EC:2.7.7.7)	0.036
A vs. E	K02003	putative ABC transport system ATP-binding protein	0.009
	K02004	putative ABC transport system permease protein	0.007
	K02030	polar amino acid transport system substrate-binding protein	0.040
	K03574	8-oxo-dGTP diphosphatase (EC:3.6.1.55)	0.029
	K15634	probable phosphoglycerate mutase (EC:5.4.2.12)	0.015
	K01462	peptide deformylase (EC:3.5.1.88)	0.019
	K03100	signal peptidase I (EC:3.4.21.89)	0.013
	K02040	phosphate transport system substrate-binding protein	0.023
	K02342	dnaQ; DNA polymerase III subunit epsilon (EC:2.7.7.7)	0.039
	K07052	uncharacterized protein	0.005
A vs. F	K02004	putative ABC transport system permease protein	0.009
	K03657	DNA helicase II / ATP-dependent DNA helicase PcrA (EC:3.6.4.12)	0.037
	K15634	probable phosphoglycerate mutase (EC:5.4.2.12)	0.038
	K04487	cysteine desulfurase (EC:2.8.1.7)	0.005
	K01462	peptide deformylase (EC:3.5.1.88)	0.029
	K03100	signal peptidase I (EC:3.4.21.89)	0.013
A vs. G	K02004	putative ABC transport system permease protein	0.045
	K03704	cold shock protein (beta-ribbon, CspA family)	0.014
	K15634	probable phosphoglycerate mutase (EC:5.4.2.12)	0.024
	K02495	oxygen-independent coproporphyrinogen III oxidase (EC:1.3.98.3)	0.012
	K01462	peptide deformylase (EC:3.5.1.88)	0.016
	K03100	signal peptidase I (EC:3.4.21.89)	0.007
	K07052	uncharacterized protein	0.030

Note: Milk samples were collected from Dezhou donkeys at 7 different lactation stages: 1 d (A), 30 d (B), 60 d (C), 90 d (D), 120 d (E), 150 d (F), and 180 d (G) after foaling.

## Data Availability

The original contributions presented in the study are included in the article; further inquiries can be directed to the corresponding author.
